# *Staphylococcus aureus* and *Staphylococcus epidermidis* Virulence Strains as Causative Agents of Persistent Infections in Breast Implants

**DOI:** 10.1371/journal.pone.0146668

**Published:** 2016-01-26

**Authors:** Daniela Chessa, Giulia Ganau, Luisella Spiga, Antonio Bulla, Vittorio Mazzarello, Gian Vittorio Campus, Salvatore Rubino

**Affiliations:** 1 Microbiology and Immunology Unit, Department of Biomedical Science, School of Medicine, University of Sassari, Sassari, Italy; 2 Plastic Surgery Unit, Department of Surgical, Microsurgical and Medical Sciences, University of Sassari, Sassari, Italy; 3 Department of Infection and Immunity, King Faisal Specialist Hospital and Research Centre, Riyadh, Saudi Arabia; University of Iowa Carver College of Medicine, UNITED STATES

## Abstract

Staphylococcus *epidermidis* and Staphylococcus *aureus* are currently considered two of the most important pathogens in nosocomial infections associated with catheters and other medical implants and are also the main contaminants of medical instruments. However because these species of Staphylococcus are part of the normal bacterial flora of human skin and mucosal surfaces, it is difficult to discern when a microbial isolate is the cause of infection or is detected on samples as a consequence of contamination. Rapid identification of invasive strains of Staphylococcus infections is crucial for correctly diagnosing and treating infections. The aim of the present study was to identify specific genes to distinguish between invasive and contaminating S. *epidermidis* and S. *aureus* strains isolated on medical devices; the majority of our samples were collected from breast prostheses. As a first step, we compared the adhesion ability of these samples with their efficacy in forming biofilms; second, we explored whether it is possible to determine if isolated pathogens were more virulent compared with international controls. In addition, this work may provide additional information on these pathogens, which are traditionally considered harmful bacteria in humans, and may increase our knowledge of virulence factors for these types of infections.

## Introduction

Implant infections have recently become a major complication related to breast and reconstructive surgery[1]. Even though surgical techniques have been modified to decrease the risk of infections, once implant infection is diagnosed, only surgical removal followed by antimicrobial chemotherapy is currently considered the mainstay of successful treatment and enables the implantation of a new device. One of the most important problems in this type of infection is the general lack of epidemiological data due to the absence of a global surveillance network of patients based on long term follow-up. Staphylococcus *epidermidis* and Staphylococcus *aureus* have become some of the most important pathogens in nosocomial infections associated with the use of catheters and other medical implants such as breast implants[2]. However because these species of Staphylococcus are part of the normal bacterial flora of human skin and mucosal surfaces, it is difficult to discern when a microbial isolate may be the cause of infection or is the result of sample contamination. S. *aureus* seems to be the most virulent pathogen that colonizes and infects both hospitalized patients with decreased immunity and healthy immunocompromised individuals[3]. As documented, S. *epidermidis* has been regarded as an innocuous commensal bacterium of human skin. Currently, this bacterium is recognized as an important human pathogen and is one of the leading causes of infections associated with the settlement of medical devices[4]. Many factors appear to contribute to the success of these types of infections, although the ability to persist as commensals and to become pathogenic is due to several virulence factors. The first breast prosthesis implantation was described in spring 1962, but only in recent years have many Staphylococcus species emerged as important pathogens with the ability to establish microbial communities on the surface of implants[5].

It has been observed that this ability to persist on medical devices is due to biofilms, mosaic polysaccharide structures consisting of microorganism agglomerations, which establish non-covalent interactions with host tissue or host proteins and are thus used to coat device surfaces[6]. The biofilm forms a heterogeneous matrix, which is able to protect bacteria from antibiotic treatment, physiologic shear, and potentially from host immune defenses[5, 7, 8]. As described in the literature, biofilm assembly proceeds in three phases as follows: (i) adhesion, (ii) proliferation/formation of the mature biofilm, and (iii) detachment/dispersal[9–11]. During the formation phase, the biofilm requires polysaccharide intercellular adhesion (PIA), which is a positively charged homopolymer of the beta-1,6-linked N-acetylglucosamine (NAG) residue[12, 13]. It was previously reported that PIA is coded by the *ica* gene cluster, which comprises the *icaA*, *icaD*, *icaB* and *icaR* genes, and it was shown that deletion of these genes may be the reason for biofilm absence[14, 15]. Importantly, *ica* expression was found to be influenced by different factors such as quorum sensing, among others; moreover, *ica* operon expression could be turned on/off by excision or insertion of IS256 and IS257 sequences[16]. Other genes may influence biofilm phenotypes or bacterium virulence and may be used as markers to distinguish between pathogenic strains and the usual microbial flora such as bap-like protein (bhp), which is a surface protein that plays an important role in improving the initial attachment of microbial cells during the first phase of biofilm formation, the osmolality resistance protein (*kdp*)[17] and the accessory gene regulator (*agr*), which is the quorum sensing system involved in the regulation of many Staphylococcus *spp* virulence genes. The microbes analyzed in this study were detected on breast prostheses implanted for aesthetic or reconstructive reasons. Some of these prostheses were explanted as a consequence of complications a few years after their placement[18]. Moreover, infections frequently lead to prosthesis removal from the same patient more than once.

The main aim of this study was to investigate whether S. *aureus* and S. *epidermidis* isolates collected from explanted breast implants had different genetic patterns compared with control samples. Furthermore, in order to study these pathogens, a new procedure was established by our group to isolate bacteria more efficiently compared with previous protocols reported in the literature. As a first step, we compared the adhesion ability of samples to their efficacy in forming biofilms; therefore, it was possible to understand if isolated pathogens were more virulent compared with international controls. Notably, we developed a rapid method to characterize strains from breast prostheses, which may be helpful for eradicating these types of infections. In addition, this work may provide additional information about these pathogens, which have traditionally been considered commensal bacteria for our organism, and may improve our knowledge of virulence factors.

## Materials and Methods

### Patients

Medical devices from patients who underwent breast implant removals for routine replacement or mechanical complications at the Plastic Surgery Unit of the University of Sassari between January 2013 and December 2014 were sent to the Microbiology Unit. All implants were collected on the day of explantation and immediately processed.

This protocol was approved by the Ethical Committee of the Azienda Sanitaria Locale di Sassari, no. 2174/CE. All patients provided informed written consent to process specimens obtained during surgery. All participant-signed forms were transmitted to and approved by the ethics committee.

### Bacterial strains and growth conditions

The bacterial strains used in this study are listed in Table 1. Eighty breast implants were explanted over a year by the Plastic Surgery Unit at the Department of Medicine and Surgery, University of Sassari and delivered on the same day to the Microbiology and Immunology Unit to be processed immediately. The breast prostheses and capsules were treated with N-acetylcysteine (Micro Biol Diagnostici, Cagliari, Italy), a mucolytic agent, and then scraped with a sterile scalpel to facilitate pathogen isolation. Staphylococcal strains were cultured in Mannitol-Salt agar (MSA) statically for two days at 37°C. All strains were subjected to the coagulase test; positive samples were classified as *S*. *aureus*, whereas the other samples were tested with standard identification systems (ID 30 Staph, BioMerieux, Firenze, Italy)[19]. Based on this biochemical test, positive samples were identified as S. *epidermidis*.

**Table 1:** List of samples.

Subspecies	Strains	Description	Source
*S*. Aureus	58		This study
*S*. Aureus	63		This study
*S*. Aureus	65		This study
*S*. Aureus	68		This study
*S*. Aureus	70		This study
*S*. Aureus	80		This study
*S*. Aureus	81		This study
*S*. Epidermidis	57		This study
*S*. Epidermidis	59		This study
*S*. Epidermidis	61		This study
*S*. Epidermidis	67		This study
*S*. Epidermidis	26		This study
*S*. Epidermidis	29		This study
*S*. Epidermidis	31		This study
*S*. Epidermidis	45		This study
*S*. Epidermidis	79		This study
*S*. Aureus	82	Control, biofilm producer	ATCC-35556
*S*. Epidermidis	83	Control, strong biofilm producer	ATCC-35984

### DNA extraction and PCR

All strains were cultured overnight in Luria Broth and then processed for DNA extraction with a classical protocol using phenol/chloroform. Briefly, all sample pellets were resuspended in 1 ml of phosphate-buffered saline 1X (PBS) and then vortexed with glass beads. Finally, the pellets were acid-washed (Sigma, Saint Louis, MO, USA) for 1 hour. The extracted DNA was used for amplification of the virulent genes reported in Table 2 [17]. The PCR protocol was as follows: preheating for 3 minutes at 94°C followed by 35 cycles of 1 minute at 94°C, 1 minute at 54°C and 2 minutes at 72°C, with a final extension at 72°C for 5 minutes. Amplified products were analyzed by 1% agarose gel electrophoresis.

**Table 2:** List of primers.

Gene	Product size (bp)	Sequence primers
*icaA* Fw	832	AAGATGTTGGCTGTGATTAC
*icaA* Rw		CAACAAGTTGAAGGCATATC
*bhp* Fw	935	TGGTATTAGGAAGCTCTCAG
*bhp* Rw		ATACCAGCGTGACGCAAATC
*kdp* Fw	813	TGGTAGCCATTCTAGGATG
*kdp* Rw		GGGATTAGGGCTCTATTTAG
*is256* Fw	762	AGTCCTTTTACGGTACAATG
*is256* Rw		TGTGCGCATCAGAAATAACG
*is257* Fw	576	CTATCTAAGATATGCATTGAG
*is257* Rw		TTAACTTGCTAGCATGATGC
*agrD* Fw	307	CATTCCTGTGCGACTTATTA
*agrD* Rw		CGTGTAATTGTGTAAATTCT

### Biofilm formation

The biofilm formation assay was performed as described by Helimann *et al* [18]. The single colony was transferred in 4 ml of trypticase soy broth supplemented with glucose (0.25% w/v) (TSB-Gluc) (Meus s.r.l., Padova, Italy) and incubated overnight at 37°C in an orbital shaker (180 rpm). Primary attachment assays were performed as follows: *S*. *aureus* and *S*. *epidermidis* strains were cultured overnight in TSB-gluc and diluted 1:100 in TSB-gluc. The bacteria were incubated until the mid-log exponential phase (OD650 = 0.8). The culture was diluted to OD650 = 0.1, and 200 ml were used to inoculate sterile 96-well polystyrene microtiter plates (Cellstar, Greiner bio-one, Kremsmünster, Austria). At 1 hour and 24 hours, the wells were gently rinsed with PBS at room temperature five times, dried in an inverted position and stained with 0.1% of crystal violet solution (Merck, Darmstadt, Germany) for 15 minutes. The wells were rinsed again, and the crystal violet solubilized in 200 ml of ethanol-acetone (80:20 v/v). The optical density at 595 nm (OD595) was determined using a microplate reader (Versa max; Molecular Devices, Sunnyvale, USA). Each assay was performed in triplicate and repeated three times. To analyze biofilm formation under flow conditions, 20 ml test tubes (Corning, Tewksbury, USA) were used with a continuous flow of 4 ml of TSB-gluc overnight. Biofilm development was recorded with a Nikon reflex D5100 camera. Two milliliters of TSB in glass test tubes were inoculated with a loopful of microorganisms from overnight culture plates and incubated for 48 hours at 37°C. Afterwards, the contents were decanted and washed with PBS (pH 7.3) and dried at room temperature. Subsequently, the tubes were stained with a 4% solution of crystal violet. Each tube was then gently rotated to ensure uniform staining, and soon after, the contents were gently decanted. The tubes were placed upside down to drain and then observed for biofilm formation, which was considered positive when visible films lined the walls and bottoms of the tubes. Ring formation at the liquid interface was not regarded as indicative of biofilm formation. The results were visually scored as follows: 0 ring-absent, 1 ring-weak, 2 rings-moderate, and 3 rings-strong[20].

### Adhesion assay

The colorectal carcinoma cell line Caco-2 (HTB-37) was obtained from the American Type Culture Collection. Caco-2 cells were grown to confluence in 24-well plates for 6 days. Prior to the adherence assays, epithelial cells were washed with ice cold PBS. Ice-cold medium was added, and the cells were infected with bacteria (10^5^ CFU/well) and allowed to adhere for 1 hour at 4°C to prevent invasion. Non-adherent bacteria were removed by 5 washes with PBS, and adherent bacteria were resuspended in PBS containing 1% (vol/vol) Triton X-100. Serial ten-fold dilutions were spread on LB agar plates to determine the number of cell-associated bacteria per well. To visualize bacterial attachment, Caco-2 cells were grown to confluence in an 8-chamber polystyrene vessel (BD FalconTM) for 5 days. The Caco-2 cells were prefixed with formalin at 2% for 30 min. After several washes with PBS, bacteria were added to the cells (10^5^ CFU/well) and incubated for 3 hours at 37°C. The chambers were washed five times and fixed with methanol for 30 seconds, followed by staining for 30 minutes with Giemsa and then examination with a light microscope[21].

### Scanning electron microscope assay

The prostheses were analyzed with a scanning electron microscope (SEM) Low Vacuum Fei Quanta 200 SEM Zeiss EVO and fixed in glutaraldehyde for 24–48 hours. Next, they were subjected to washes in PBS 1X to remove excess fixative. At this point, the prostheses were dehydrated by passages in acetone solutions with increasing concentrations (25%, 50%, 75%, 95% and total) and then dried with hexamethyldisilazane (HMDS) to remove fluid from the samples. Finally, the samples were subjected to a preliminary procedure of surface coating with metallic elements and were analyzed using the scanning electron microscope.

### Statistical analysis

A parametric test (paired Student’s *t* test) was used to calculate whether differences in the fold increases of biofilm production expression among the different strains were statistically significant. A two-tailed *p* value of < 0.05 was considered significant.

## Results

### Virulence genes

First, all strains were subjected to the coagulase test; the positive samples were classified as S. *aureus*, whereas the other samples were tested by standard identification systems. Samples positive for this biochemical test were identified as S. *epidermidis*. Moreover the utilization of N-acetylcysteine as a treatment is a novel protocol, which allows for faster and more effective isolation of samples and may serve as a prompt and rapid diagnostic method to support clinician diagnoses. To observe the genetic differences among all the pathogens isolated in this study, chromosomal DNA extraction was performed to analyze the presence of virulence genes. It was previously noted that most virulence genes such as *ica* may be used to show correlations between infectious and noninfectious strains of Staphylococcus *spp*.[22]. First, we determined the *ica*A distributions in the samples by analytical PCR. IcaA was found in 63% of the S. *epidermidis* strains and 35% of the S. *aureus* strains, which differed from the results of the control samples (Tables 3 and 4). In addition, the frequency of the presence of other important genes was analyzed; *bhp* was found in 36% of the S. *epidermidis* isolates; *kdp* was found in 28% of the S. *epidermidis* strains and was not recovered in the S. *aureus* strains. The peptide precursor AgrD was more positive in the S. *aureus* isolates (86%) compared with the S. *epidermidis* (46%) isolates. The insertion of IS256 and IS257 sequences was positive in all bacteria isolated. These findings suggest the high variability of the presence of these virulence genes, as well as the importance of studying genes in order to complete a fingerprinting analysis that may help to assess different risk levels.

**Table 3:** PCR results showing Staphylococcus *aureus* strains.

Samples	*icaA*	*bhp*	*kdp*	*is256*	*is257*	*agrD*
58	-	+	-	+	+	-
63	-	-	-	+	+	+
65	-	-	-	+	+	+
68	-	-	-	+	+	-
70	-	-	-	+	+	+
80	+	-	-	+	+	+
81	+	-	-	+	+	+
82	+	+	+	+	+	-

**Table 4:** PCR results showing Staphylococcus *epidermidis* strains.

Samples	*icaA*	*bhp*	*kdp*	*is256*	*is257*	*agrD*
57	-	+	-	+	+	-
59	+	-	-	+	+	+
61	+	-	-	+	+	+
67	-	+	+	+	+	-
26	+	+	-	+	+	-
29	+	+	-	-	+	-
31	+	-	-	+	+	-
45	+	-	-	+	+	-
79	+	-	-	+	+	+
83	-	+	+	+	+	-

### Biofilm formation

**Fig 1:**
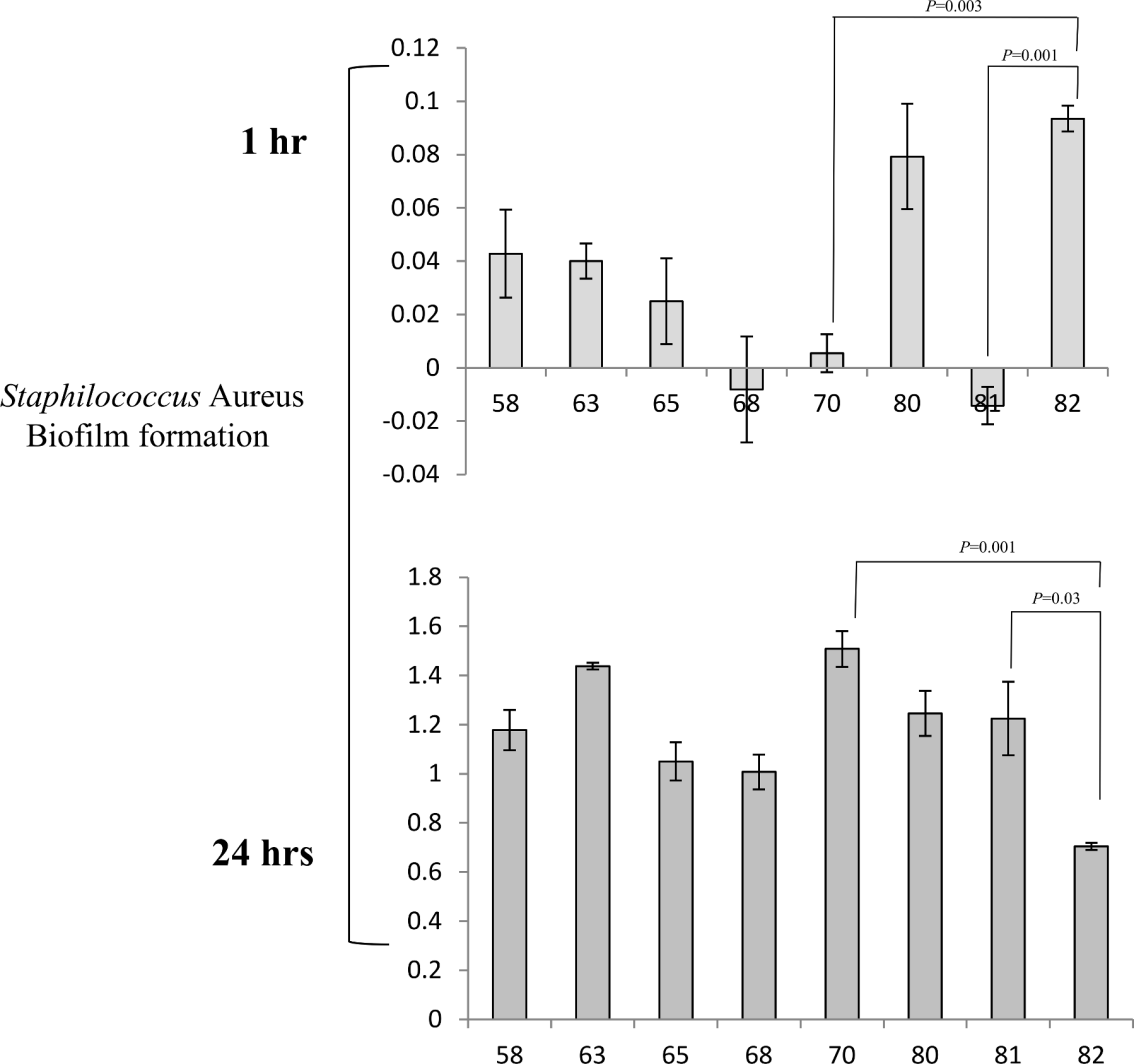
Detection of biofilm formation in Staphylococcus *aureus* strains. All strains were incubated for 1 hour and 24 hours in TSB-gluc and absorbance was detected at 595 nm using a microplate reader. All data are shown as geometric means from three independent experiments with standard deviations.

**Fig 2:**
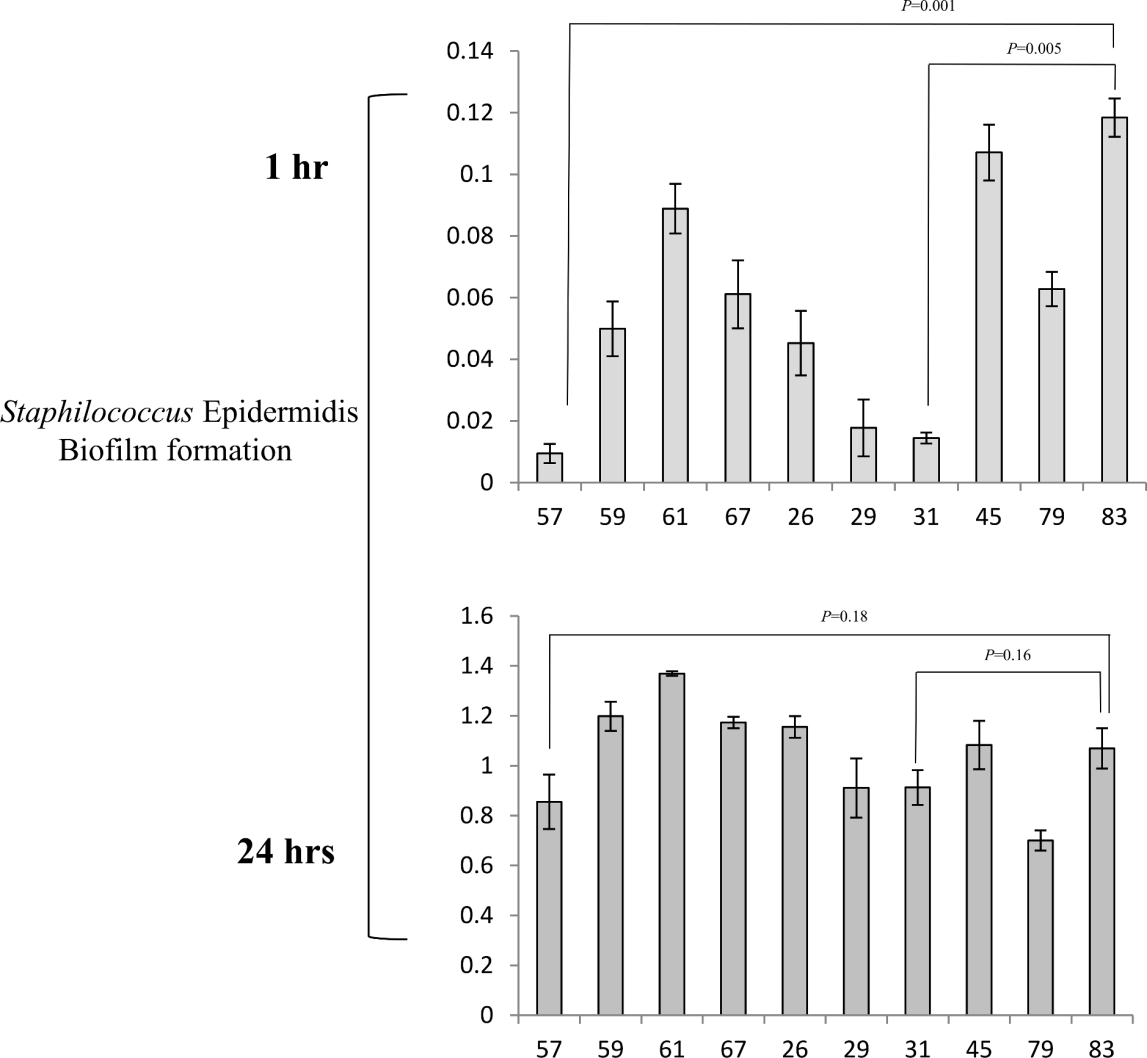
Detection of biofilm formation in Staphylococcus *epidermidis* strains. All strains were incubated for 1 hour and 24 hours in TSB-gluc, and absorbance was detected at 595 nm using a microplate reader. All data are shown as geometric means from three independent experiments with standard deviations.

**Fig 3:**
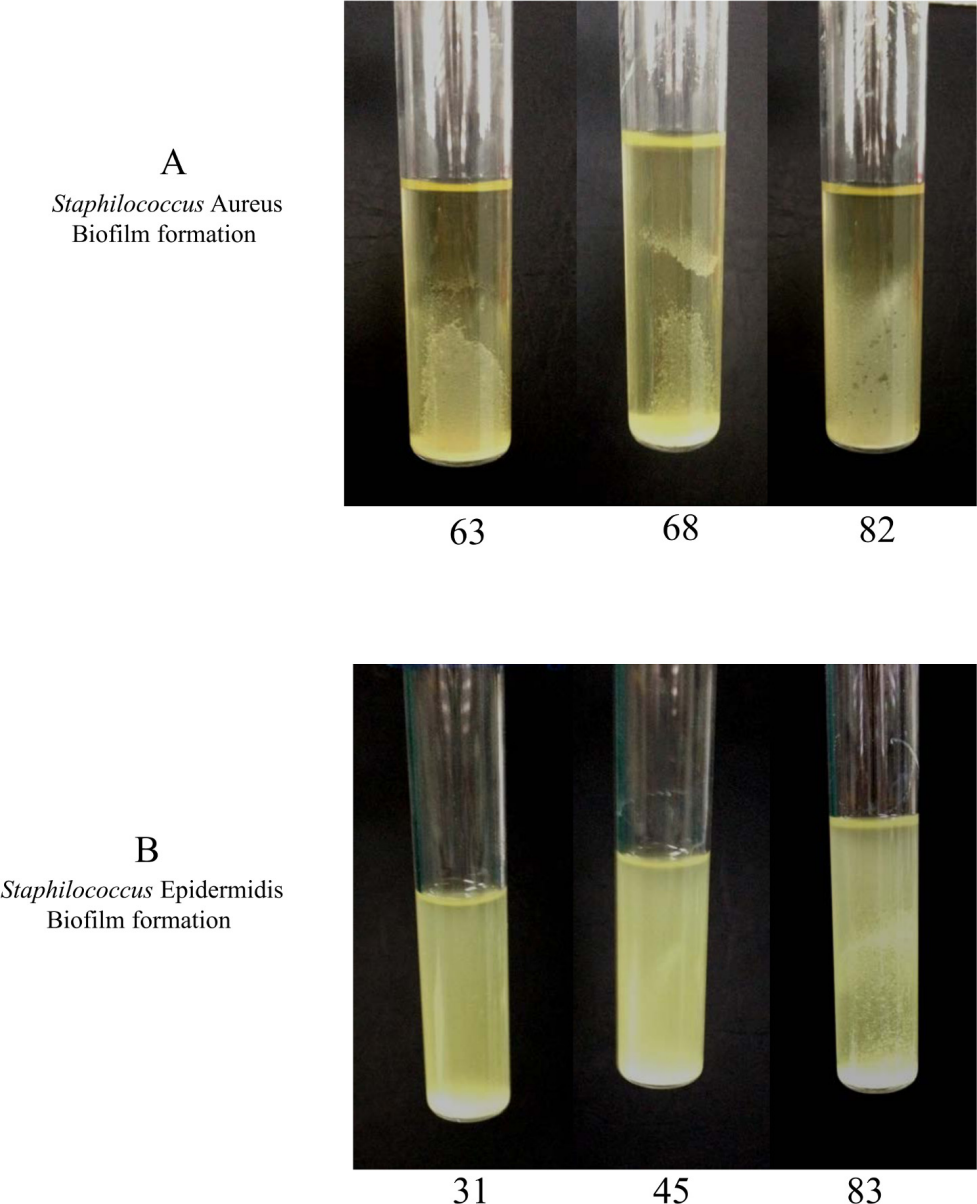
Biofilm formation in glass vials. (A) Biofilm formation from Staphylococcus *aureus* strains in glass vials with TSB-gluc after overnight incubation. (B) Biofilm formation from Staphylococcus *epidermidis* strains in glass vials with TSB-gluc after overnight incubation.

**Fig 4:**
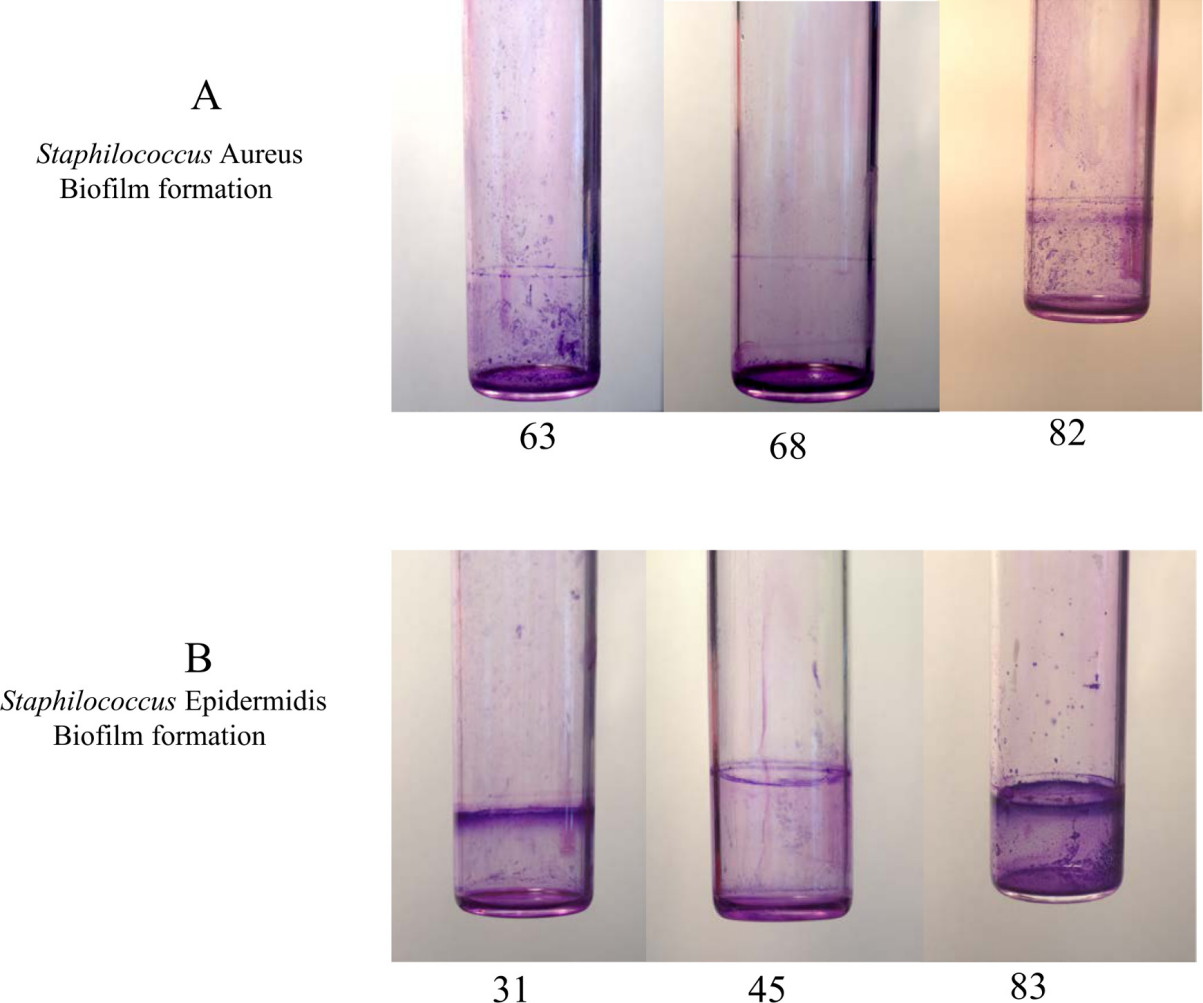
Ring test to evaluate biofilm formation. (A) Biofilm formation in Staphylococcus *aureus* based on ring test in glass vials using crystal violet solution; sample number 82 is the ATCC control sample. (B) Biofilm formation in Staphylococcus *epidermidis* based on ring test in glass vials using crystal violet solution; sample number 83 is the ATCC control sample.

Based on the general agreement that biofilms are the basis for persistent or chronic bacterial infections, we tried to assess the capability of our isolates to form biofilms. Different techniques were used to address this issue. First, bacteria were cultured overnight for biofilm quantification, and absorbance was determined using a microplate reader at 595 nm. S. *aureus* showed a strong phenotype in biofilm production; in this experiment, the control strain was a moderate biofilm producer. After the first hour of incubation, the S. *aureus* strains were weak producers of biofilm compared with the controls (p = 0.003); at 24 hours of incubation, all samples became stronger producers of biofilm (p = 0.001) compared with the controls, which behaved as moderate biofilm producers (Fig 1). Taken together, these results demonstrate that our isolates became strong producers of biofilm after a long incubation, which gave them the ability to persist for a long period of time. The same procedure was repeated using S. *epidermidis*, showed a high variability in biofilm. In fact, all strains isolated from human breast prostheses after an incubation time of 1 hour showed divergent biofilm production ranging from strong to weak compared with our control strains, which were classified as strong producers. Indeed, significant differences were observed (p < 0.05) among most of the strains relative to the controls. After incubation for 24 hours, all strains became strong producers of biofilm, and unexpectedly, some of the strains exceeded the biofilm production of the highly positive controls (Fig 2). Notably, these results showed that strains subjected to a long incubation period exhibited an improved ability to form biofilms. Our findings are intriguing as they suggest the presence of a strong virulence factor, which influences the ability of different strains to form biofilms and helps these strains to develop as nosocomial infectious agents. Furthermore, this phenotype may play a role in limiting the effectiveness of antibiotic therapy and may be an obstacle in bacterial recognition by the innate immune response system during persistence in the host. To investigate biofilm production, we cultured isolates under flow conditions overnight; the outcome of this culture is represented in Fig 3. Some of the samples that formed biofilms can be observed. To confirm our data, we tried to estimate the ability to form biofilms using a 4% solution of crystal violet in tubes in which strains were cultured overnight. Biofilm formation was observed for all strains, and as shown by the rings on the walls of the vials, all strains were strong biofilm producers (Fig 4).

### Adhesion assay

**Fig 5:**
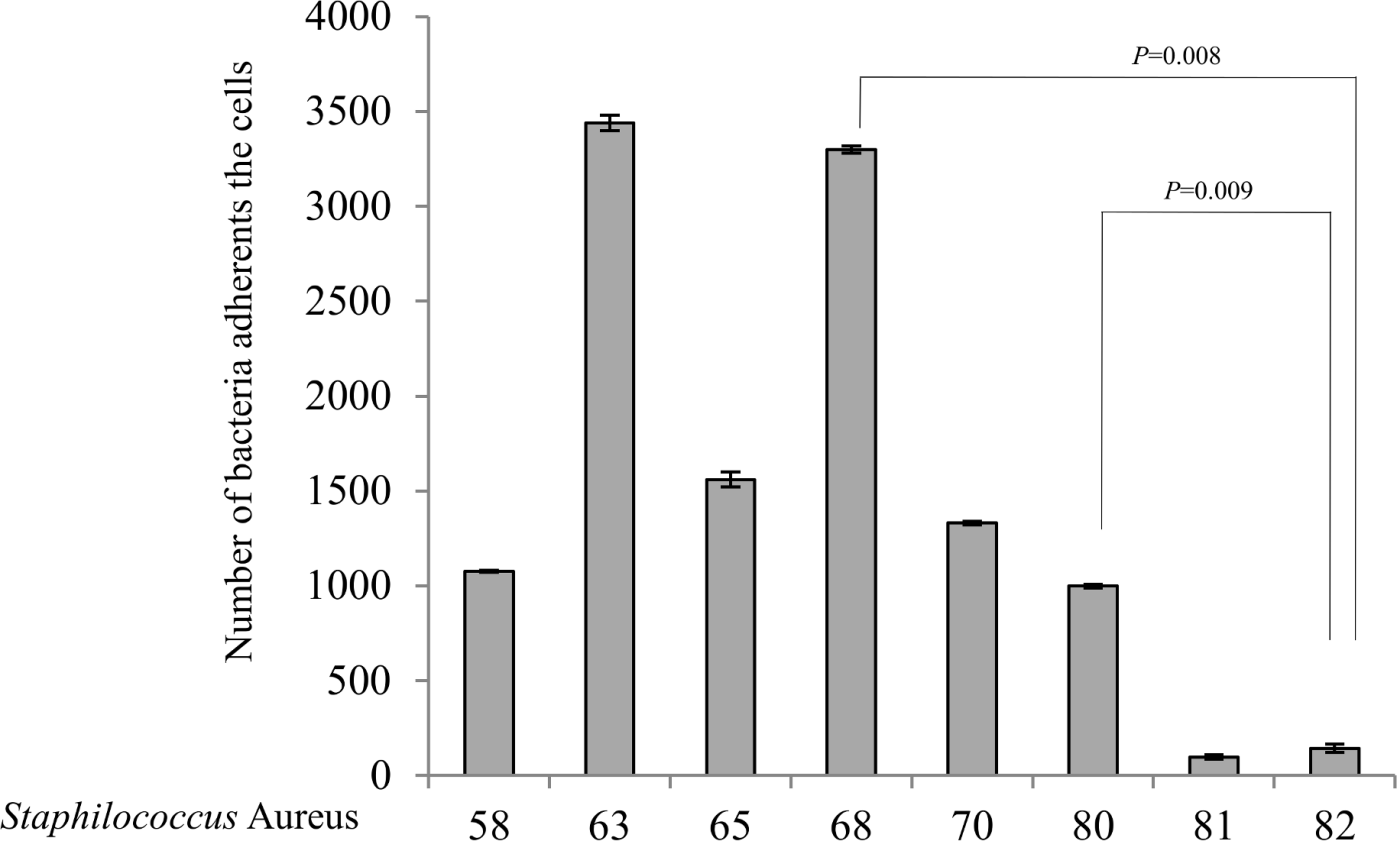
Binding of Staphylococcus *aureus* to live human colonic epithelial cells at 4°C to prevent bacterial invasion. All data are shown as the average of cell-associated bacteria ± standard error from four independent experiments. Statistical significance of the differences is indicated in brackets.

**Fig 6:**
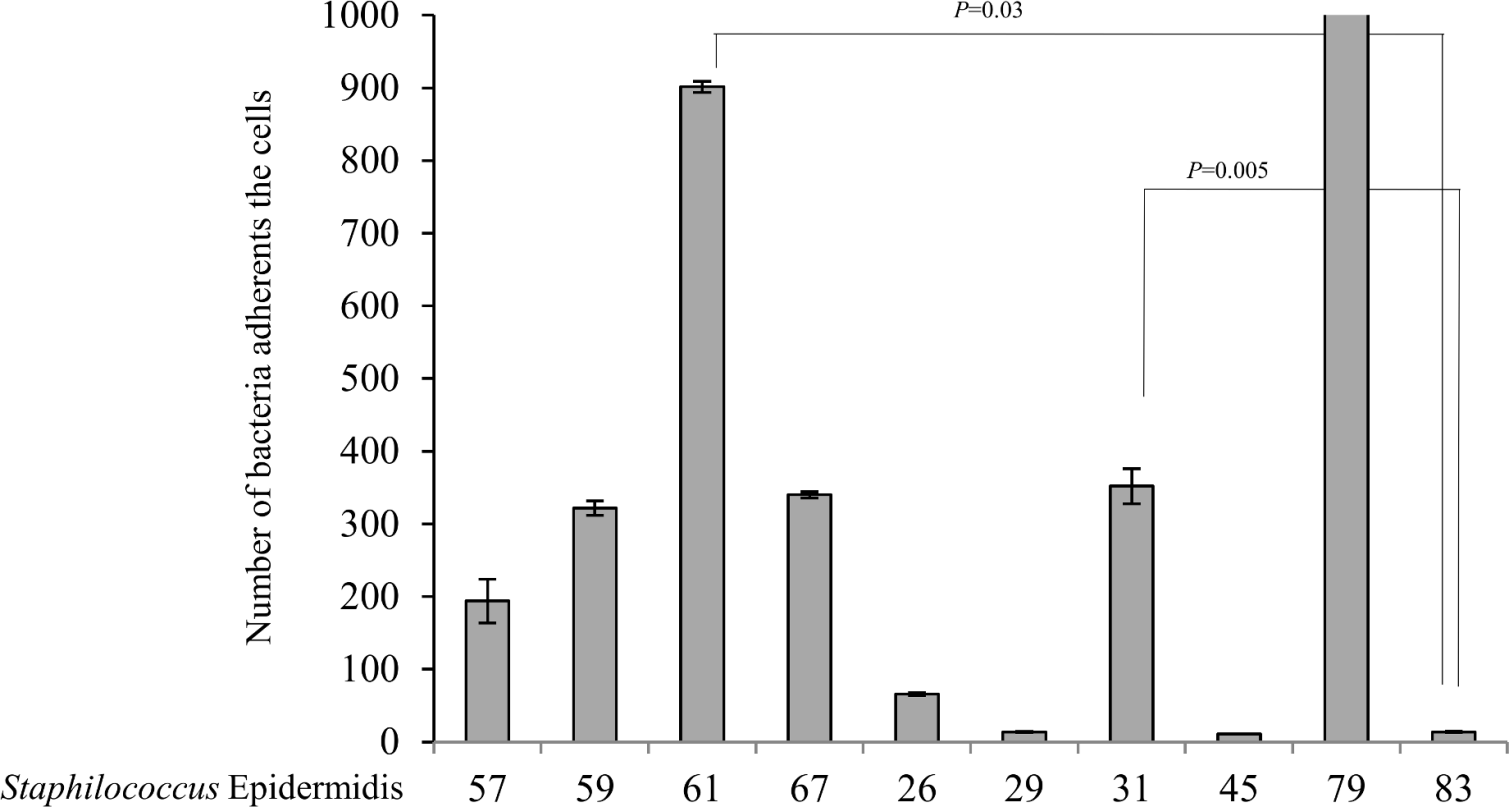
Binding of Staphylococcus *epidermidis* to live human colonic epithelial cells at 4°C to prevent bacterial invasion. All data are shown the as average of cell-associated bacteria ± standard error from four independent experiments. Statistical significance of the differences is indicated in brackets.

**Fig 7:**
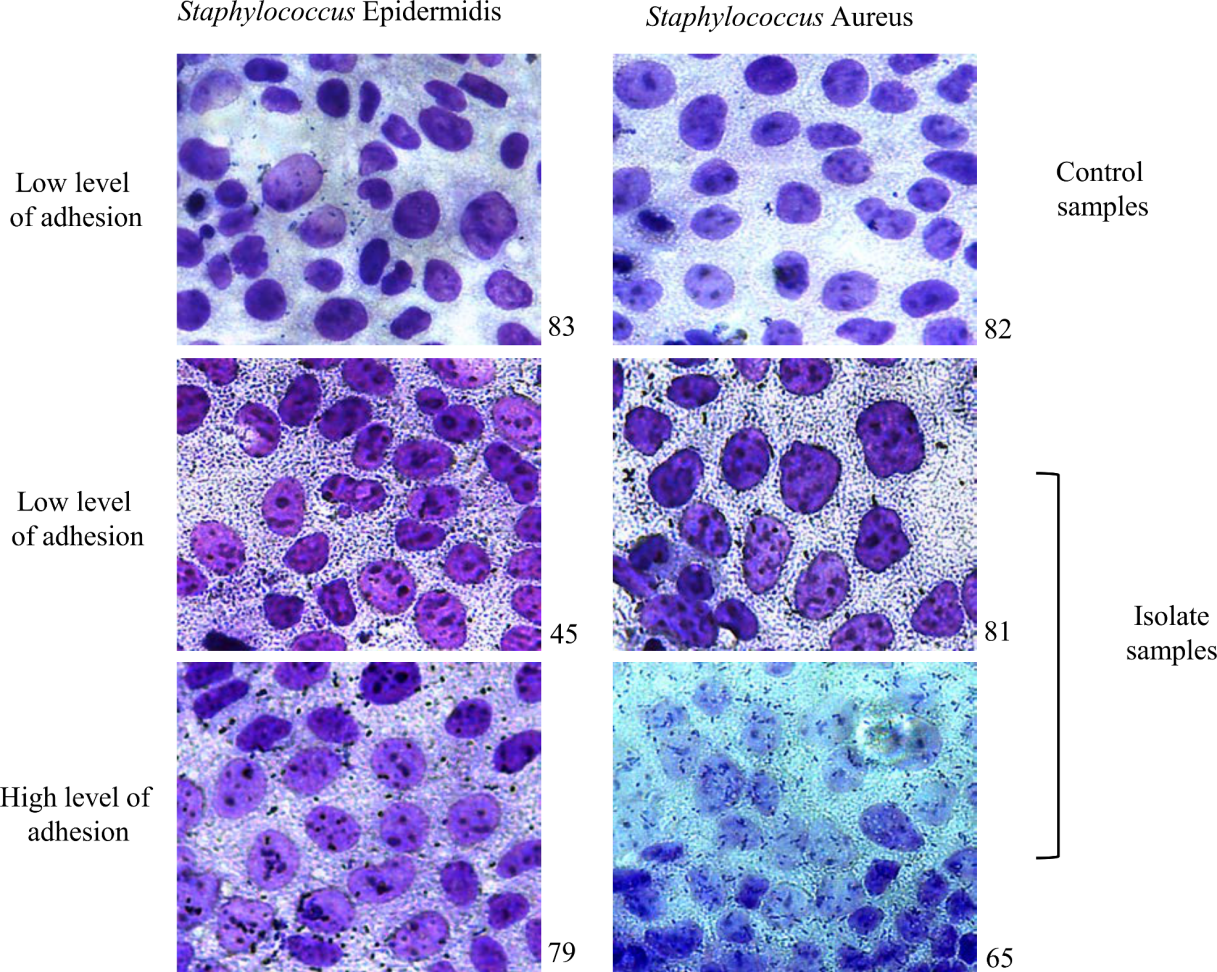
Visualization of bacterial attachment to formalin fixed Caco-2 cells using Giemsa staining.

Adhesion is the first step towards infection; however, if bacteria are not able to provide a surface interaction, the specific diseases caused by these bacteria will not develop. This important phenotype may be a discriminating factor between pathogens and commensal bacteria. To study this characteristic in strains isolated during our studies, we performed an adhesion assay using the colorectal carcinoma cell line Caco-2. After a cold incubation to prevent bacterial invasion, the strains were added to the cells, and it was possible to count only bacteria that had the capability to adhere to cell surfaces. Seventy-five percent of the S. *aureus* showed an ability to adhere to Caco-2 cells, which was more than the percentage of positive controls showing this ability (p = 0.009) (Fig 5). Unremarkably, the same phenotype was discovered in S. *epidermidis*: 54% of these strains were able to attach to Caco-2 cell surfaces (p = 0.005) (Fig 6). The binding of S. *aureus* and S. *epidermidis* to formalin fixed Caco-2 cells was observed and differed from the binding of these bacteria to the controls (sample numbers 82 and 83, Fig 7). All samples had different adhesion graduations ranging from low to high; thus, these data are correlated with the observations presented in Figs 5 and 6. These findings underline the importance of studying different virulence factors in all strains able to cause disease in humans because the ability of these strains to become pathogenic is related to their capacity to express different phenotypes compared with control samples. Our experiments showed how these isolates may establish strong interactions with cell surfaces and may expose cells to a higher risk of host invasion.

### Scanning electron microscope visualization

**Fig 8:**
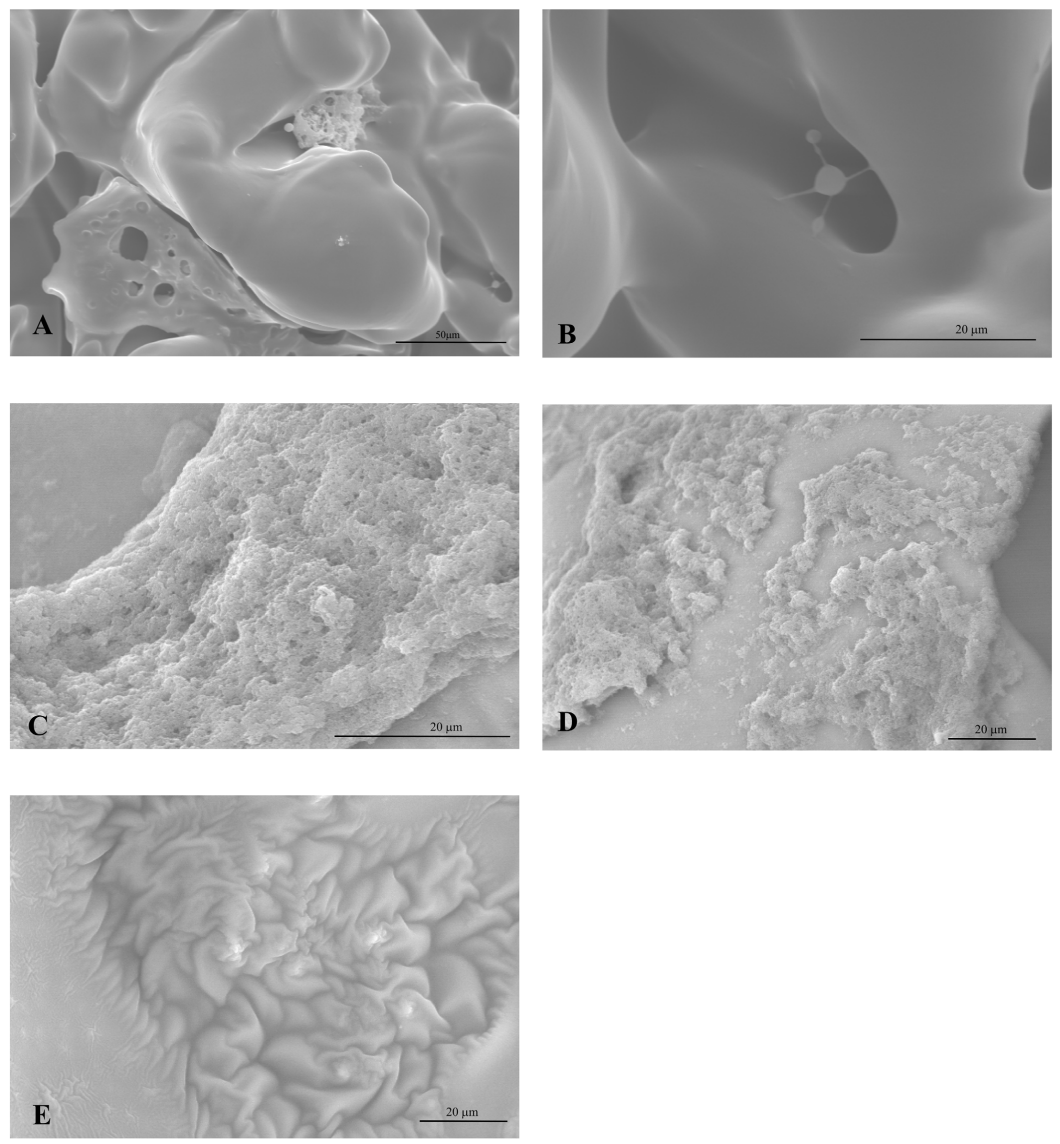
Breast prostheses analyzed by scanning electron microscope. Letters (A) magnification 2000X, (B), (C), (D) represent different images of breast prostheses in which biofilm formation can be observed with 4000X of magnification. (E) Negative control breast prostheses without any traces of biofilm formation.

To confirm our findings, the prostheses were analyzed by a scanning electron microscope. The samples were fixed in glutaraldehyde, and after several washes, were observed through the scanning electron microscope. No additive difference was observed in the prostheses positive for Staphylococcus as it was found that they had also been covered by biofilm (Fig 8C and 8D). In addition, the prostheses negative for Staphylococcus and other pathogens were clean and did not show any biofilm formation (Fig 8E). Moreover, we observed that a strain that was starting to attach to the prosthesis surface (Fig 8B). Furthermore, these experiments gave us the opportunity to see how medical devices are covered by biofilms, and this information may be of a great help in understanding why it was not possible to treat these infections with normal antimicrobial therapy.

## Conclusions

S. *aureus* and S. *epidermidis* are related species and share the same potential virulence factors necessary to establish infections on medical devices[23]. This important characteristic is due to different virulence factors that seem to be more prevalent in some serotypes compared with control bacteria. Biofilm formation has been recognized during recent years as an extremely important factor contributing to the virulence of pathogenic bacteria in chronic infections. In fact, biofilms represent a surface-attached agglomeration of cells commonly embedded in a heterogeneous matrix[5, 24, 25]. To study these phenotypes, we were able to observe how isolates from breast prostheses act as strong biofilm producers compared with moderate and strong producers in control samples. Thus, we were able to underline the presence of an important trait in isolated samples because biofilms are also responsible for the persistence of Staphylococcus infections on medical device surfaces. This is a crucial finding as it is estimated that over 60% of treated infections in developing countries originate from biofilm formation[26]. Clinical presentations in patients do not always provide a clear framework for diagnoses, but the presence of fever and leukocytosis associated with edema and swelling should suggest the possibility of an infection[1]. It has been demonstrated that under physiological *in vitro* conditions, leukocytes attach to, penetrate, and produce cytokines in response to mature S. *aureus* biofilms. Similar results have been observed with other bacteria, indicating that biofilm formation associated with persistent infections may also cause chronic inflammation[27]. Specifically, our results indicate that S. *aureus* and S. *epidermidis* isolates also have another significant phenotype: adhesion. Approximately 67.5% of the strains isolated in this study showed this capability, suggesting how these isolates create strong interactions with cell surfaces and have more opportunities for host invasion. Furthermore, our study suggests that the characterizations of these types of strain may represent a promising approach to prevent development of infections in breast implants; our hypothesis is that these strains may be part of normal patient epidermal flora, and future investigations are warranted to determine infection routes. Importantly, these findings have significantly expanded our knowledge of the existence of different virulence factors in look-alike strains with diverse infectious potential that can be used to discriminate common bacteria from pathogenic bacteria.
